# Variability in published rates of influenza-associated hospitalizations: A systematic review, 2007-2018

**DOI:** 10.7189/jogh.10.020430

**Published:** 2020-12

**Authors:** Katherine M Roguski, Melissa A Rolfes, Jeremy S Reich, Zachary Owens, Neha Patel, Julia Fitzner, Vanessa Cozza, Kathryn E Lafond, Eduardo Azziz-Baumgartner, A Danielle Iuliano

**Affiliations:** 1 US Centers for Disease Control and Prevention (CDC), Atlanta, Georgia, USA; 2 Emory University, Rollins School of Public Health, Department of Epidemiology, Atlanta, Georgia, USA; 3 World Health Organization, Global Influenza Programme, Geneva, Switzerland

## Abstract

**Background:**

Influenza burden estimates help provide evidence to support influenza prevention and control programs at local and international levels.

**Methods:**

Through a systematic review, we aimed to identify all published articles estimating rates of influenza-associated hospitalizations, describe methods and data sources used, and identify regions of the world where estimates are still lacking. We evaluated study heterogeneity to determine if we could pool published rates to generate global estimates of influenza-associated hospitalization.

**Results:**

We identified 98 published articles estimating influenza-associated hospitalization rates from 2007-2018. Most articles (65%) identified were from high-income countries, with 34 of those (53%) presenting estimates from the United States. While we identified fewer publications (18%) from low- and lower-middle-income countries, 50% of those were published from 2015-2018, suggesting an increase in publications from lower-income countries in recent years. Eighty percent (n = 78) used a multiplier approach. Regression modelling techniques were only used with data from upper-middle or high-income countries where hospital administrative data was available. We identified variability in the methods, case definitions, and data sources used, including 91 different age groups and 11 different categories of case definitions. Due to the high observed heterogeneity across articles (*I^2^*>99%), we were unable to pool published estimates.

**Conclusions:**

The variety of methods, data sources, and case definitions adapted locally suggests that the current literature cannot be synthesized to generate global estimates of influenza-associated hospitalization burden.

Seasonal influenza epidemics are important contributors to the global burden of hospitalizations each year [1]. The World Health Organization’s (WHO) Pandemic Influenza Preparedness Partnership Contribution Implementation Plan 2013-2016 called for a better understanding of influenza disease burden globally, especially in low-income and tropical climate countries [2]. Quantifying influenza disease burden at a national, regional, or global level may help countries understand the relative importance of influenza as a public health priority, identify potential target groups for influenza prevention and control strategies, and explore the cost benefit of these interventions [3,4].

Improvements in laboratory and surveillance capacity in many middle- and low-income countries since the 2009 influenza A(H1N1) pandemic have allowed countries to better understand influenza virus transmission within their population and collect the requisite data to estimate influenza-associated disease burden [5]. In 2015, the WHO published guidance to leverage hospital-based influenza surveillance data to estimate rates of influenza-associated hospitalizations [6]. These guidelines focus on a multiplier approach. This approach, in brief, estimates influenza-associated hospitalization by multiplying counts of hospitalizations by the portion of samples from sentinel surveillance testing positive for influenza viruses on a weekly, monthly, or annual level then summed across the year. The approach was designed for use with severe acute respiratory infection (SARI) surveillance data often collected in low income hospital settings following WHO guidelines [7]. An adjustment to the population denominator can be used when the number of hospitalizations collected does not represent all of the hospitalizations occurring within the population, such as when hospitalizations are only captured from one hospital but three exist in the region. An alternative method is based on regression modelling, which estimates excess influenza-associated hospitalizations and has commonly been used for estimating excess deaths due to influenza [8-10]. The regression modelling approach often requires a greater number of years of data with a larger sample size than the multiplier approach in order for the models to converge [10]. In both approaches, researchers use a variety of data sources and adjustments to generate their estimates of influenza-associated hospitalization at the local level [11-13].

Through a systematic review, we aimed to identify all published articles estimating rates of influenza-associated hospitalizations, describe methods and data sources used, and identify regions of the world where estimates are still lacking. We evaluated study heterogeneity to determine if we could pool the current literature to generate global estimates of influenza-associated hospitalization. This review will inform future efforts to generate global estimates of influenza-associated hospitalization burden.

## METHODS

### Search strategy and extraction

We systematically searched six computerized literature databases (Medline, Embase, Cumulative Index to Nursing and Allied Health Literature [CINAHL], Cochrane Library, Global Health, and Literatura Latino-Americana e do Caribe em Ciências da Saúde [LILACS]) to identify articles published from January 1, 2007 - February 28, 2018. As one of our exclusion criteria for laboratory testing was the exclusion of publications using laboratory tests other than reverse transcription polymerase chain reaction (RT-PCR), we limited our search window to articles published in 2007 or later, as RT-PCR testing was rarely used before 2007 [11]. Search terms included “influenza,” “burden,” “hospitalization,” and “disease incidence,” and we did not limit results by language or place of publication. Details of the search strategy used are presented in Table S1 in the **Online Supplementary Document**.

### Article selection

Once we removed duplicate publications, two independent reviewers screened titles and abstracts for potential exclusion based on the criteria presented in **Table 1**. We reviewed full text articles for all remaining titles and abstracts and any with discrepant reviews. Two independent reviewers evaluated English and Spanish full text articles using ranked exclusion criteria detailed in **Table 1**. A native speaker reviewed non-English and non-Spanish articles, summarized each article, and responded to questions from two reviewers who independently evaluated each article. A third reviewer was asked to review any articles with discrepant decisions about exclusion and all three reviewers discussed the article until the discrepancy was resolved. We looked for additional publications by reviewing references of reviewed articles with influenza-associated hospitalization rates.

**Table 1:** Exclusion criteria for title and abstract and full text review

Title and abstract exclusion criteria (not ranked)	Full text exclusion criteria (ranked)
Only presented data on non-human infections of influenza	Only presented data on non-human infections of influenza
Only presented data on human infections of avian, swine, or novel influenza	Article focused on a particular patient or case study
Article focused on a particular case study	For non-modelling papers, no influenza testing performed
For non-modelling articles, no influenza testing performed	No data on hospitalized patients presented
No data on hospitalized patients presented	No methods for estimating catchment population specified for hospital
No methods for estimating catchment population specified for hospital	No estimates of influenza-associated hospitalization rates presented
If laboratory testing for influenza performed, no confirmation using reverse transcription polymerase chain reaction (RT-PCR)	If laboratory testing for influenza performed, no confirmation using RT-PCR
Less than 12 months of data (or one full influenza season) presented	Less than 12 months of data (or one full influenza season) presented
Estimates or data only available during 2009 pandemic period for influenza A(H1N1)pdm09	Estimates or data only available during 2009 pandemic period for influenza A(H1N1)pdm09
Estimates only presented for special populations or risk groups (eg, pregnant women, health care workers)	Estimates only presented for special populations or risk groups (eg, pregnant women, health care workers)
Only mortality estimates presented	Only mortality estimates presented

### Analysis

For accepted articles, we used a standardized tool to extract data for analysis, including information about country, region, or sites where data were collected, case definitions, data sources, estimation methods, influenza virus laboratory testing methods, and rates of influenza-associated hospitalizations. When available, we also obtained confidence intervals (CIs) or other reported measures of error for rates of influenza-associated hospitalization. We classified age group-specific rates into the age grouping that most closely matched eight age groups used by WHO (all ages, <2 years, 2-4 years, <5 years, 5-14 years, 15-49 years, 50-64 years, and ≥65 years) [6]. We categorized methods used to estimate influenza-associated hospitalization rates into either multiplier or regression modelling approach. For regression models, we further noted whether the model included covariates for laboratory confirmed influenza virus circulation. We also categorized the method used to identify hospitalized patients as either: symptom-based identification through logbook, chart review, or patient interview, or diagnosis-based identification using administrative databases using International Classification of Disease codes. We captured the case definition used by the study to define their rate numerator and summarized these case definitions into 11 categories (Table S2 in the **Online Supplementary Document**). All extracted rates were population-based rates, where article authors defined a catchment population of hospital study sites to use as their rate denominator. We extracted any information on methods used to adjust catchment populations, such as health care utilization or hospital administrative surveys.

We assessed study heterogeneity to determine if rates could be logically pooled using meta-analysis. For this quantitative analysis, we included only a subset of the full data set: all nonzero, nonnegative rate estimates with a reported measure of error. We excluded zero and negative rates because the log rate variance was too great for the models to run. Only overall influenza-associated hospitalization rates were included; influenza type- or subtype-specific estimates were excluded. We used a three-level random effects meta-regression model to account for within article clustering. Study heterogeneity was assessed statistically using the Cochran Q test and the *I^2^* statistic [14]. As the heterogeneity between articles remained high (*I*^2^>75%), we were unable to complete a meta-analysis to generate pooled summary estimates [14]. Analyses were completed using R software 3.5.1 (R Foundation for Statistical Computing, Vienna, Austria) and Stata 13.1 (StataCorp, College Station, TX, USA).

## RESULTS

### Article selection

**Figure 1:**
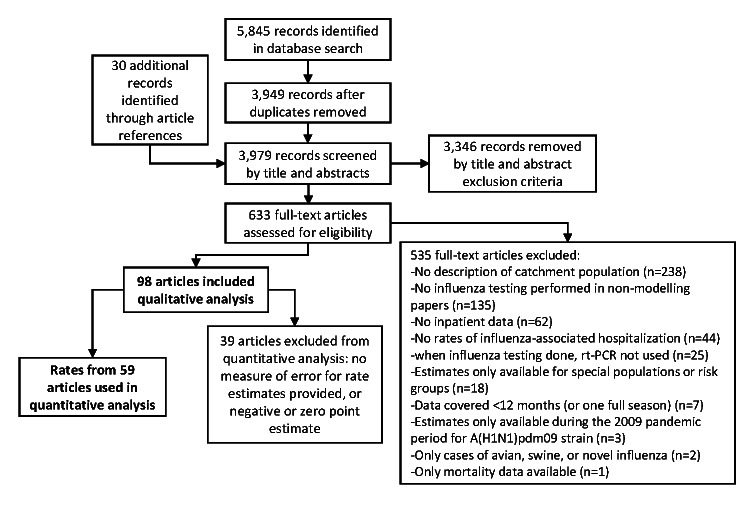
Identification and selection of eligible articles for the systematic review of rates of influenza-associated hospitalization.

Our database search identified 3949 articles of potential interest, and we identified 30 additional articles through the review of relevant bibliographies (**Figure 1**). After we reviewed titles and abstracts, we excluded 3346 articles. Of the 633 articles not excluded during the title and abstract review, we accepted 98 for data extraction. We most frequently excluded articles in the full-text review for not using and defining a catchment population for hospitalizations (44%, n = 238/535) or for not performing influenza testing in non-modelling articles (25%, 135/535). Details of the articles included in this review are presented in Table S3 in the **Online Supplementary Document**.

### Article methods and data sources

**Figure 2:**
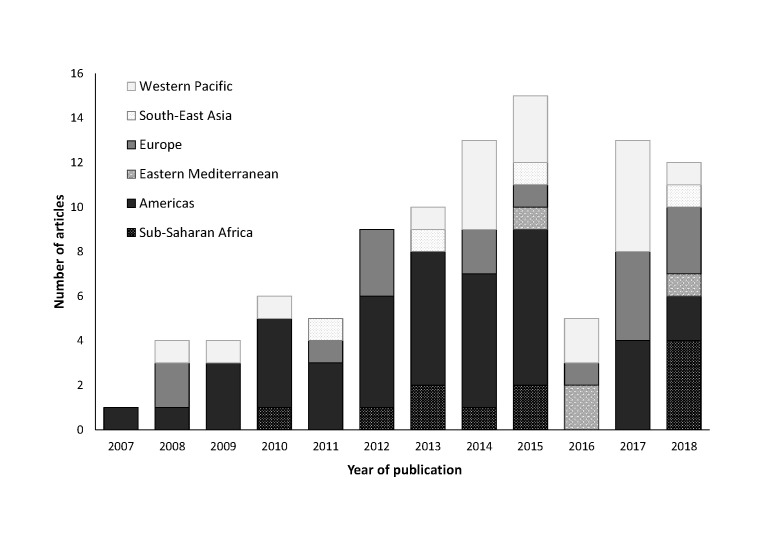
Distribution of extracted articles by publication year and World Health Organization region.

Of the 98 extracted articles, 2867 estimates of influenza-associated hospitalization rates were available from 39 countries (representing 64% of the global population) [15]. The majority of articles (n = 78, 80%) used a multiplier approach to generate influenza-associated hospitalization rates (**Table 2**). Nineteen (19%) used a variety of regression modelling techniques, such as Poisson (n = 5, 26%), linear (n = 4, 21%), and negative binomial (n = 8, 42%) regression. Of the articles using regression methods, two did not include influenza covariates in their models and each used a different model type (linear regression and autoregressive integrated moving average [ARIMA] models) [16,17]. Across the 98 extracted articles, 91 different age groups were used. Fifteen extracted articles were published from 2007-2010, 52 from 2011-2015, and 31 from 2016-February 2018 (**Figure 2**).

**Table 2:** Characteristics of articles meeting inclusion criteria

	Multiplier approach			Regression model		
**Variable**	Number (%) of articles (n = 78)*	**Number of rates**	**Number of countries**	Number (%) of articles (n = 19)*	**Number of rates**	**Number of countries**
World Bank income grouping:						
High	48 (62)	686	12	15 (79)	972	8
Upper-middle	11 (14)	184	7	4 (21)	67	2
Lower-middle	9 (12)	329	6	0 (0)	–	–
Low	9 (12)	612	4	0 (0)	–	–
World Health Organization region:						
Sub-Saharan Africa	11 (14)	672	4	0 (0)	–	–
Americas	35 (45)	478	5	7 (37)	693	3
Eastern Mediterranean	4 (5)	108	3	0 (0)	–	–
Europe	11 (14)	135	6	5 (26)	164	3
South-East Asia	4 (5)	148	3	0 (0)	–	–
Western Pacific	12 (15)	270	8	7 (37)	182	4
Case definition:†						
ARI with fever	24 (31)	884	18	0 (0)	–	–
ARI, fever not required	18 (23)	271	9	3 (16)	41	3
Pneumonia	9 (12)	140	6	0 (0)	–	–
Influenza	34 (44)	421	8	2 (11)	97	2
Pneumonia and influenza	1 (1)	35	1	12 (63)	252	10
Respiratory	0 (0)	–	–	9 (47)	170	5
Circulatory	0 (0)	–	–	3 (16)	31	3
Respiratory and circulatory	0 (0)	–	–	5 (26)	400	4
Acute medical illness	2 (3)	61	1	1 (5)	9	1
All cause	0 (0)	–	–	2 (11)	14	2
Sepsis	0 (0)	–	–	1 (5)	4	1
Case-patient identification method:						
Symptom-based identification	43 (55)	1,311	25	0 (0)	–	–
Administrative database	35 (45)	501	9	19 (100)	1,039	10
Denominator adjustment:						
None	12 (15)	221	9	3 (16)	88	2
Hospital administrative survey	13 (17)	221	9	0 (0)	–	–
Healthcare utilization survey	9 (12)	654	7	0 (0)	–	–
Complete population coverage	42 (54)	656	14	15 (79)	832	8

ARI – acute respiratory infection

*Total percentages may add to more than 100%, as some articles may contain results that fall into multiple categories.

†See Table S2 in the **Online Supplementary Document** for detailed case definitions.

Sixty-four articles (65%) presented rates from high-income countries, as defined by the World Bank, whereas 18 (18%) presented rates from lower-middle-income or low-income countries [18]. All 18 articles presenting rates from lower-middle- or low-income countries used a multiplier approach (**Table 2**). Six of the nine articles with rates from low-income countries presented data from Kenya [19-24]. The majority of extracted articles using either a multiplier approach or regression modelling techniques (n = 46/78, 59% and n = 12/19, 63%, respectively) presented rates from countries in the Americas or Europe. Thirty-four of the articles overall (35%) included estimates from the United States. All articles presenting rates from countries in the Sub-Saharan Africa, Eastern Mediterranean, and South-East Asia regions used a multiplier approach.

All extracted articles presented a variety of different case definitions or discharge diagnoses to identify potential influenza-associated hospitalizations. Thirty-six articles (37%) defined hospitalizations based on laboratory-confirmation of influenza or a discharge diagnosis of influenza; the vast majority of these (n = 34/36) used a multiplier approach. Forty-one articles (42%) used a case definition of hospitalized acute respiratory infection (ARI); 20 focused on ARI with fever, 17 on ARI without a fever requirement, and four using both ARI with fever and ARI without fever case definitions. Certain case definitions, such as all respiratory, all circulatory, combined respiratory and circulatory, or all-cause diagnoses, which represent broad categories used to code administrative data, were only used in articles using regression modelling techniques. Overall, 17 (17%) articles estimated influenza-associated hospitalization rates using more than one case definition. Of these, nine (53%) articles used regression modelling techniques where authors compared estimates across different case definitions (eg, all respiratory, all circulatory, and pneumonia and influenza diagnostic codes). The remaining eight articles (47%) used multiplier techniques with different case definitions for different age groups (n = 8) or different years (n = 1) following WHO case definitions [25]. While we focused on 11 categories of case definitions for this review, there were many small differences between individual articles within each of these categories. For example, we identified eleven different definitions of ARI requiring fever and fourteen additional definitions of ARI without the fever requirement (Table S2 in the **Online Supplementary Document**).

All 19 articles that used regression modelling techniques identified hospitalizations using administrative databases. Forty-five percent (n = 35) of articles using a multiplier approach identified hospitalizations using administrative databases and all were describing high and upper-middle-income countries. Fifty-seven (58%) articles noted that the hospitalizations identified represented all hospitalizations in their study region (complete population coverage). All articles using a health care utilization survey (n = 9) or a hospital administrative survey (n = 13) to adjust the population denominator used a multiplier approach.

### Extracted rates

**Figure 3:**
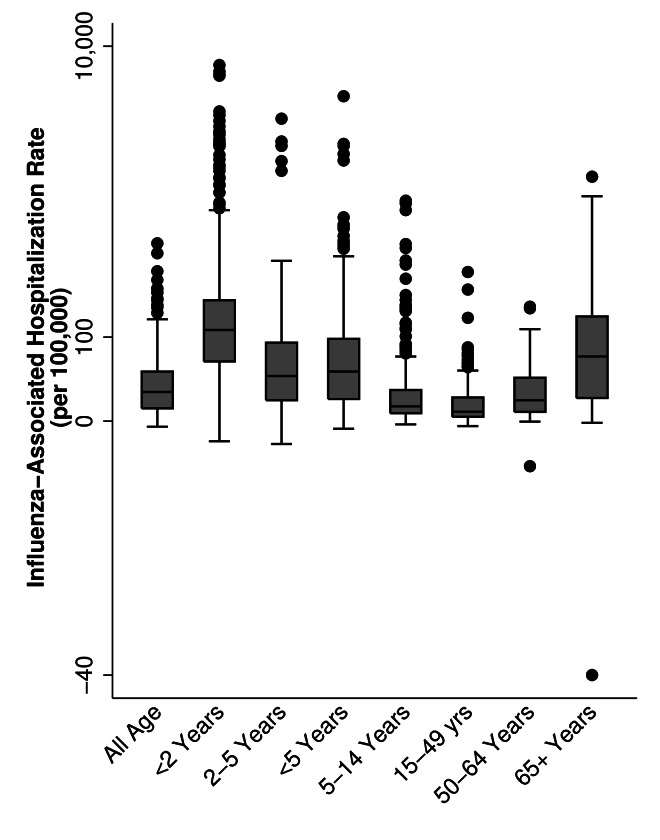
Published population-based rates of influenza-associated hospitalization, by combined age groups.

**Figure 4:**
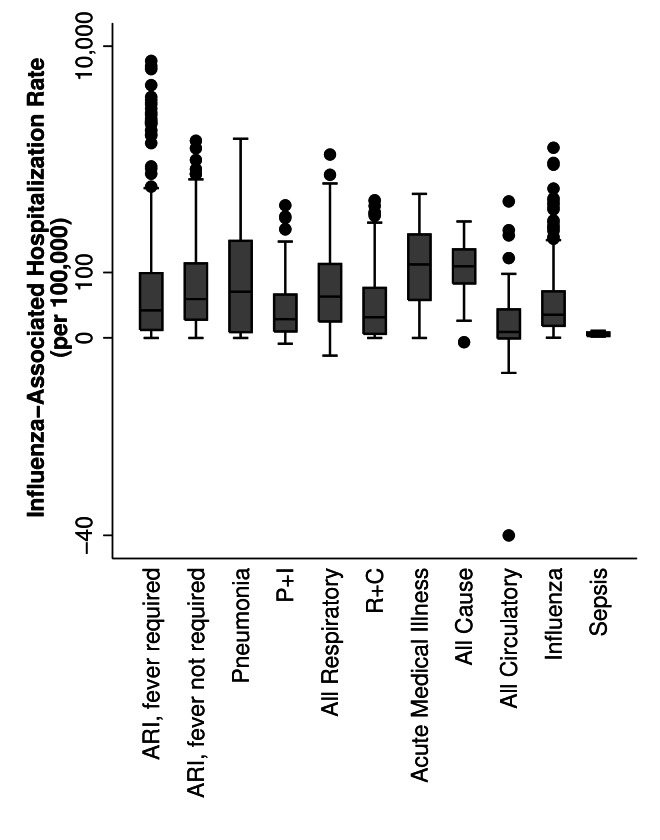
Published population-based rates of influenza-associated hospitalization, by case definition categories. ARI – acute respiratory illness, P+I – pneumonia and Influenza, R+C – respiratory and circulatory.

A median of three years of data were presented in each article (interquartile range: 2-6 years), with estimates from 1991-2017. We observed variability in rates of influenza-associated hospitalizations by age group, region, and method. Across all study years, annual rates, per 100 000 population, ranged from -11 to 7543 for children aged <2 years, -12 to 3417 for children aged 2-4 years, -4 to 4763 for children aged <5 years, -2 to 999 for children aged 5-14 years, -3 to 325 for persons aged 15-49 years, -20 to 179 for persons aged 50-64 years, and -40 to 1437 for persons aged ≥65 years (**Figure 3**). There were 17 published annual influenza-associated hospitalization rates, from four articles [8,9,26,27], that were negative using regression models; of which, 53% were estimated rates of influenza-associated circulatory hospitalizations. By case definition, influenza-associated all-cause hospitalizations and influenza-associated acute medical illness hospitalizations had the highest median rates (118 and 123 per 100 000 respectively); the highest individual rate (7543 per 100 000) resulted from an estimate where hospitalizations were defined as of ARI requiring fever (**Figure 4**). Overall, annual rates using regression modelling were more tightly grouped (mean: 57.8; 95% CI: 51.6, 63.9 per 100 000) compared to those using a multiplier approach (mean: 134.3; 95% CI: 112.7, 155.9 per 100 000) (Figure S1 in the **Online Supplementary Document**).

Out of all extracted rates, 928 (35% of 2687) had no reported measure of error, an additional 12 had negative point estimates, and 109 rates were zero. These rates were all excluded from our evaluation of heterogeneity. After limiting the quantitative analysis to non-type or subtype specific (all influenza) estimates, 1500 rates from 54 articles remained. The Cochran Q test indicated the presence of heterogeneity (*Q =* 1.3x10^7^, *P* < 0.001), and the *I*^2^ statistic attributed 99.97% of the variance in the estimates to study heterogeneity rather than chance. Therefore, we were unable to generate pooled summary estimates or conduct a meta-analysis. Even after stratifying the data by age group, method classification (multiplier vs regression modelling approach), case-patient identification method, World Bank income classification, or World Health Organization region, study heterogeneity remained high within each sub-group (>99%) (Table S4 in the **Online Supplementary Document**).

## DISCUSSION

In recent years, there has been an increased focus and support for countries to understand the influenza burden in their populations and these efforts have contributed to a need to understand influenza burden at the global level, as well. In our review, we identified 39 countries that have estimated influenza-associated hospitalization burden in their populations, with the majority of articles being published from 2014-2018. However, we also found substantial variability in these published rates, as well as, the approaches, age groups, case definitions, data sources, and other factors being reported. Because of this large heterogeneity in published hospitalization rates, generating pooled estimates from the current literature was not possible.

Among the articles we reviewed, two main methodological approaches were used: regression modelling and multiplier techniques. Regression modelling methods require robust hospitalization data sets with a number of years of data [10]. In our review, articles using regression modelling techniques were limited to high-income countries with access to hospital administrative databases. Because of the use of administrative data, many of these articles used broader case definitions, such as all respiratory or all respiratory and circulatory causes, to capture a more complete picture of influenza-associated hospitalization burden [28,29]. In most lower-income countries, however, such administrative databases do not exist or are poorly maintained [30]. In all articles from lower-income countries identified in our review, case-patients were identified based on a specific syndrome, such as acute respiratory illness with or without fever, which may be more specific and less sensitive than the broader case definitions used in the regression modelling articles. Without access to robust administrative databases or the ability to capture case-patients with broader case definitions in lower-income countries, it is challenging to compare modelled hospitalization rates for high-income countries directly to hospitalization rate estimates calculated using specific case definitions from lower-income countries.

Among the regression modelling articles, substantial heterogeneity existed among the published hospitalizations rates, perhaps driven by the differences the model inputs and assumptions. Only two publications used regression modelling techniques without influenza laboratory data and each used a different model (ARIMA [17] and linear regression [16]). We also observed differences in how authors handled negative model outputs. Negative estimates of influenza-associated hospitalization can occur with regression models when the number of predicted hospitalizations for a given week are less than the estimated baseline number of hospitalizations based on seasonal patterns of influenza activity and hospitalization numbers. In some studies, these negative values were replaced with zero [29], while other studies maintained the original values produced by the model [9]. Some authors also included additional covariates into their models to help reduce internal variability, such as respiratory syncytial virus circulation [31], public holidays [8], weekly mean temperature [32], and weekly mean relative humidity [32]. Any of these adjustments may have influenced individual estimates, adding to the observed heterogeneity.

Estimates generated using a multiplier technique were even more variable than those generated using regression modelling methods. While the WHO published guidelines for estimating influenza disease burden using a multiplier approach in 2015 [6], many articles were published before the guidelines were published or interpreted the guidelines differently [30]. For example, some articles analyzed monthly [33] or annual [34,35] data. Some incorporated adjustment factors to account for testing practices and influenza diagnostic test type [12], whereas others did not. Some adjusted their estimates to account for potential admissions in hospitals in the surrounding area [30], whereas others did not. Additionally, articles using multiplier techniques used either administrative databases or symptom-based patient identification depending on available data in a country.

We also observed a high variability in published rates among articles using certain case definitions, such as laboratory-confirmed influenza. This variability may be in part due to differences in testing practices: differences in determining which patients are tested for influenza viruses, including per-protocol following uniformly applied case definitions or relying on clinician discretion, and the use of different influenza diagnostics tests [12,36]. While using a case definition of laboratory-confirmed influenza hospitalizations may be most specific, this approach may actually underestimate true influenza burden if few hospitalized patients with influenza illness are tested [9,37]. County-specific studies looking at practitioner testing practices could be useful to create an adjustment factor to account for this under reporting and variability, such as the adjustment used by researchers in the United States [12]. On the other hand, removing the condition for laboratory confirmation and attributing hospitalizations to influenza based on a broader case definition risks inflating the influenza-associated hospitalization estimates [38]. Additional studies are needed to explore the range of diagnoses and complications among individuals hospitalized with laboratory-confirmed influenza.

We observed that, during the past decade, more countries have published estimates of influenza-associated hospitalization burden. Despite this trend, the majority of articles identified in this study were from the United States and other high-income countries. In recent years, lower- or middle-income countries are beginning to report on burden estimates, as many have improved and expanded their surveillance capacity for influenza [5]. *Influenza and Other Respiratory Viruses* published a supplemental issue focused on estimates of influenza burden in 2018 that highlights this improvement in capacity [39]. Among the supplemental articles, of which 11 were included in this review, seven were from countries that had not previously published burden estimates. Additionally, the regional WHO offices in the Eastern Mediterranean and Western Pacific regions also published specific supplements, in 2016 and 2018, respectively, for influenza disease burden estimates [40,41]. While the growing literature is encouraging, we hope to see continued efforts to pursue and publish consistent estimates of seasonal influenza burden, specifically from under-represented areas like the Eastern Mediterranean and South-East Asia regions.

We excluded many articles during the full text review because the authors were not able to estimate the hospitals’ catchment population (denominator) precluding them from being able to generate population-based rate estimates. This may have limited the number of countries included in this study. To address this limitation, WHO published an approach in 2015 to assist countries conducting influenza virus sentinel surveillance to estimate hospital catchment populations [6]. While only five extracted articles [30,34,42-44] in this analysis used the WHO approach, a number of additional articles have been published after our search period from countries that have previously not published estimates of influenza-associated hospitalization burden [45-47].

While we were not able to pool results from our systematic review of the literature, published estimates of global influenza-associated hospitalization burden do exist. Wang X et al. [48] and The Global Burden of Disease 2017 Influenza Collaborators [49] have published global estimates of influenza-associated acute lower respiratory infection hospitalizations in children <5 years and influenza lower respiratory tract infection hospitalizations, respectively. Both groups use a combination of published and unpublished country-level data to model burden focusing on a specific outcome measure, lower respiratory tract infections. While these estimates provide one interpretation of influenza hospitalization burden, they do not account for alternative case definitions (outcomes) or methods for estimating influenza hospitalization burden that are used globally and we identified in our systematic review.

Like all systematic reviews, the data on case definition and methodology we were able to capture was limited by the information provided in each publication. Many articles published influenza-associated hospitalization rates as a secondary objective of their study and often did not include detailed methods or measures of error for their estimated rates. We made an effort to extract additional information from references and reach out to authors directly, however, lack of information may have limited our review. Additionally, a wide variety of age groups and case definitions were used across publications, which limited direct comparisons. While these age groups or case definitions may be relevant to the local context, the variability between articles within each category made it challenging to categorize accepted articles. Finally, we were not able to capture and account for additional factors that may have impacted published estimates, such as population health, care seeking behaviors, access to care, hospitalization practices, laboratory specimen collection and handling, and the application of case definitions. The variability in determining if a patient should be hospitalized independent of disease severity, such as bed capacity, admitting physician, and ability to pay, could increase unmeasured variability in hospitalization burden estimates compared to similar death burden estimates.

In conclusion, there is growing literature on seasonal influenza-associated hospitalization burden, though estimates from lower-middle- and low-income countries are still limited. Published influenza-associated hospitalizations rates were estimated using a variety of methods, data sources, and case definitions, which complicates the comparison and synthesis of these data on a global level. Due to the strong correlation between methodologic characteristics and high variability across estimates, we were unable to generate pooled estimates from the current published literature. Future collaborations and analyses between countries using standardized methods and case definitions across different populations or analyses applying different methods in the same study population could allow for a direct comparison of the impact of specific epidemiologic methods and help to understand the differences in rates of influenza-associated hospitalization.

## Additional material

Online Supplementary Document

## References

[1] Lafond KE Nair H Rasooly MH Valente F Booy R Rahman M Global Role and Burden of Influenza in Pediatric Respiratory Hospitalizations, 1982-2012: A Systematic Analysis. PLoS Med. 2016;13:e1001977. 10.1371/journal.pmed.1001977 27011229 PMC4807087

[2] World Health Organization. Pandemic Influenza Preparedness Framework: Partnership Contribution Implementation Plan 2013-2016 Geneva, Switzerland: WHO, 2015.

[3] Cromer D van Hoek AJ Jit M Edmunds WJ Fleming D Miller E The burden of influenza in England by age and clinical risk group: a statistical analysis to inform vaccine policy. J Infect. 2014;68:363-71. 10.1016/j.jinf.2013.11.013 24291062

[4] Peasah SK Azziz-Baumgartner E Breese J Meltzer MI Widdowson MA Influenza cost and cost-effectiveness studies globally–a review. Vaccine. 2013;31:5339-48. 10.1016/j.vaccine.2013.09.013 24055351

[5] Polansky LS Outin-Blenman S Moen AC Improved Global Capacity for Influenza Surveillance. Emerg Infect Dis. 2016;22:993-1001. 10.3201/eid2206.151521 27192395 PMC4880096

[6] World Health Organization. A Manual for Estimating Disease Burden Associated with Seasonal Influenza. Geneva, Switzerland: WHO; 2015.

[7] World Health Organization. Global Epidemiological Surveillance Standards for Influenza. 2014.

[8] Wu P Presanis AM Bond HS Lau EHY Fang VJ Cowling BJ A joint analysis of influenza-associated hospitalizations and mortality in Hong Kong, 1998-2013. Sci Rep. 2017;7:929. 10.1038/s41598-017-01021-x 28428558 PMC5430505

[9] Schanzer DL Saboui M Lee L Nwosu A Bancej C Burden of influenza, respiratory syncytial virus, and other respiratory viruses and the completeness of respiratory viral identification among respiratory inpatients, Canada, 2003-2014. Influenza Other Respir Viruses. 2018;12:113-21. 10.1111/irv.12497 29243369 PMC5818333

[10] Thompson WW Weintraub E Dhankhar P Cheng PY Brammer L Meltzer MI Estimates of US influenza-associated deaths made using four different methods. Influenza Other Respir Viruses. 2009;3:37-49. 10.1111/j.1750-2659.2009.00073.x 19453440 PMC4986622

[11] Millman AJ Reed C Kirley PD Aragon D Meek J Farley MM Improving Accuracy of Influenza-Associated Hospitalization Rate Estimates. Emerg Infect Dis. 2015;21:1595-601. 10.3201/eid2109.141665 26292017 PMC4550134

[12] Reed C Chaves SS Daily Kirley P Emerson R Aragon D Hancock EB Estimating influenza disease burden from population-based surveillance data in the United States. PLoS One. 2015;10:e0118369. 10.1371/journal.pone.0118369 25738736 PMC4349859

[13] Thompson WW Shay DK Weintraub E Brammer L Bridges CB Cox NJ Influenza-associated hospitalizations in the United States. JAMA. 2004;292:1333-40. 10.1001/jama.292.11.1333 15367555

[14] Higgins JPT Thompson SG Deeks JJ Altman DG Measuring inconsistency in meta-analyses. BMJ. 2003;327:557-60. 10.1136/bmj.327.7414.557 12958120 PMC192859

[15] World Population Prospects. The 2015 Revision. 2015. Available: http://esa.un.org/unpd/wpp/Download/Standard/Population/. Accessed: 15 December 2015.

[16] Azziz-Baumgartner E Cabrera AM Cheng PY Garcia E Kusznierz G Calli R Incidence of influenza-associated mortality and hospitalizations in Argentina during 2002-2009. Influenza Other Respir Viruses. 2013;7:710-7. 10.1111/irv.12022 23210456 PMC5855154

[17] Rodrigues E Machado A Silva S Nunes B Excess pneumonia and influenza hospitalizations associated with influenza epidemics in Portugal from season 1998/1999 to 2014/2015. Influenza Other Respir Viruses. 2018;12:153-60. 10.1111/irv.12501 29460423 PMC5818339

[18] The World Bank. Country and Lending Groups. 2015. Available: http://data.worldbank.org/about/country-and-lending-groups. Accessed: 15 December 2015.

[19] Berkley JA Munywoki P Ngama M Kazungu S Abwao J Bett A Viral etiology of severe pneumonia among Kenyan infants and children. JAMA. 2010;303:2051-7. 10.1001/jama.2010.675 20501927 PMC2968755

[20] Dawa JA Chaves SS Nyawanda B Njuguna HN Makokha C Otieno NA National burden of hospitalized and non-hospitalized influenza-associated severe acute respiratory illness in Kenya, 2012-2014. Influenza Other Respir Viruses. 2018;12:30-7. 10.1111/irv.12488 29243402 PMC5818348

[21] Emukule GO Khagayi S McMorrow ML Ochola R Otieno N Widdowson MA The burden of influenza and RSV among inpatients and outpatients in rural western Kenya, 2009-2012. PLoS One. 2014;9:e105543. 10.1371/journal.pone.0105543 25133576 PMC4136876

[22] Feikin DR Ope MO Aura B Fuller JA Gikunju S Vulule J The population-based burden of influenza-associated hospitalization in rural western Kenya, 2007-2009. Bull World Health Organ. 2012;90:256-263A. 10.2471/BLT.11.094326 22511821 PMC3324867

[23] Fuller JA Summers A Katz MA Lindblade KA Njuguna H Arvelo W Estimation of the national disease burden of influenza-associated severe acute respiratory illness in Kenya and Guatemala: a novel methodology. PLoS One. 2013;8:e56882. 10.1371/journal.pone.0056882 23573177 PMC3584100

[24] McMorrow ML Emukule GO Njuguna HN Bigogo G Montgomery JM Nyawanda B The Unrecognized Burden of Influenza in Young Kenyan Children, 2008-2012. PLoS One. 2015;10:e0138272. 10.1371/journal.pone.0138272 26379030 PMC4574572

[25] Fitzner J Qasmieh S Mounts AW Alexander B Besselaar T Briand S Revision of clinical case definitions: influenza-like illness and severe acute respiratory infection. Bull World Health Organ. 2018;96:122-8. 10.2471/BLT.17.194514 29403115 PMC5791775

[26] Goldstein E Greene SK Olson DR Hanage WP Lipsitch M Estimating the hospitalization burden associated with influenza and respiratory syncytial virus in New York City, 2003–2011. Influenza Other Respir Viruses. 2015;9:225-33. 10.1111/irv.12325 25980600 PMC4548992

[27] Khieu TQ Pierse N Telfar-Barnard LF Huang QS Baker MG Estimating the contribution of influenza to hospitalisations in New Zealand from 1994 to 2008. Vaccine. 2015;33:4087-92. 10.1016/j.vaccine.2015.06.080 26143611

[28] Warren-Gash C Blackburn R Whitaker H McMenamin J Hayward AC Laboratory-confirmed respiratory infections as triggers for acute myocardial infarction and stroke: a self-controlled case series analysis of national linked datasets from Scotland. Eur Respir J. 2018;51:1701794. 10.1183/13993003.01794-2017 29563170 PMC5898931

[29] Ang LW Yap J Lee V Chng WQ Jaufeerally FR Lam CS Influenza-Associated Hospitalizations for Cardiovascular Diseases in the Tropics. Am J Epidemiol. 2017;186:202-9. 10.1093/aje/kwx001 28338806

[30] Stewart RJ Ly S Sar B Ieng V Heng S Sim K Using a hospital admission survey to estimate the burden of influenza-associated severe acute respiratory infection in one province of Cambodia—methods used and lessons learned. Influenza Other Respir Viruses. 2018;12:104-12. 10.1111/irv.12489 29453796 PMC5818350

[31] Matias G Taylor RJ Haguinet F Schuck-Paim C Lustig RL Fleming DM Modelling estimates of age-specific influenza-related hospitalisation and mortality in the United Kingdom. BMC Public Health. 2016;16:481. 10.1186/s12889-016-3128-4 27278794 PMC4898386

[32] Ang LW Lim C Lee VJ Ma S Tiong WW Ooi PL Influenza-associated hospitalizations, Singapore, 2004-2008 and 2010-2012. Emerg Infect Dis. 2014;20:1652-60. 10.3201/eid2010.131768 25275710 PMC4193272

[33] Abdel-Hady DM Al Balushi RM Al Abri BA Al Abri SS Al Kindi HS Al-Jardani AK Estimating the burden of influenza-associated hospitalization and deaths in Oman (2012-2015). Influenza Other Respir Viruses. 2018;12:146-52. 10.1111/irv.12500 29205882 PMC5818336

[34] Susilarini NK Haryanto E Praptiningsih CY Mangiri A Kipuw N Tarya I Estimated incidence of influenza-associated severe acute respiratory infections in Indonesia, 2013-2016. Influenza Other Respir Viruses. 2018;12:81-7. 10.1111/irv.12496 29205865 PMC5818340

[35] Nyamusore J Rukelibuga J Mutagoma M Muhire A Kabanda A Williams T The national burden of influenza-associated severe acute respiratory illness hospitalization in Rwanda, 2012-2014. Influenza Other Respir Viruses. 2018;12:38-45. 10.1111/irv.12494 29197152 PMC5818355

[36] Bundy DG Strouse JJ Casella JF Miller MR Burden of influenza-related hospitalizations among children with sickle cell disease. Pediatrics. 2010;125:234-43. 10.1542/peds.2009-1465 20100764 PMC3283164

[37] Grijalva CG Weinberg GA Bennett NM Staat MA Craig AS Dupont WD Estimating the undetected burden of influenza hospitalizations in children. Epidemiol Infect. 2007;135:951-8. 10.1017/S095026880600762X 17156502 PMC2870647

[38] Tempia S Walaza S Moyes J Cohen AL McMorrow ML Treurnicht FK Quantifying how Different Clinical Presentations, Levels of Severity and Healthcare Attendance Shape the Burden of Influenza-Associated Illness: a Modeling Study from South Africa. Clin Infect Dis. 2019;69:1036-48. 10.1093/cid/ciy1017 30508065 PMC7804385

[39] Special Issue:Influenza Disease Burden. Influenza Other Respir Viruses. 2018;12:1-192. Available: https://onlinelibrary.wiley.com/toc/17502659/2018/12/1. Accessed: 23 October 2020.

[40] Mandil A Bresee J Tageldin MA Azad TM Khan W Research agenda on persistent and unpredictable threat of influenza and emerging respiratory infections: a public health necessity in the Eastern Mediterranean Region. East Mediterr Health J. 2016;22:430-1. 10.26719/2016.22.7.430 27714735

[41] Crooks K Massey PD Taylor K Miller A Campbell S Andrews R Planning for and responding to pandemic influenza emergencies: it's time to listen to, prioritize and privilege Aboriginal perspectives. Western Pac Surveill Response J. 2018;9:5-7. 31832246 10.5365/wpsar.2018.9.5.005 PMC6902653

[42] Gouya M Rezaei F Haghdoost A Nabavi M Farahi KS Mostafavi E Estimation of influenza and severe acute respiratory illness incidence (burden) in three provinces of the Islamic Republic of Iran, 2012 and 2013. East Mediterr Health J. 2016;22:432-9. 10.26719/2016.22.7.432 27714736

[43] Gefenaite G Pistol A Popescu R Popovici O Ciurea D Dolk C Estimating burden of influenza-associated influenza-like illness and severe acute respiratory infection at public healthcare facilities in Romania during the 2011/12-2015/16 influenza seasons. Influenza Other Respir Viruses. 2018;12:183-92. 10.1111/irv.12525 29144598 PMC5818344

[44] Zhang Y Li C Tang YQ Zhao XJ Liu ZC Pan Y Estimating the burden of influenza-associated hospitalization for cases of severe acute respiratory infection, Beijing, 2015. [In Chinese] Zhonghua Yu Fang Yi Xue Za Zhi. 2017;51:1097-101. 29262491 10.3760/cma.j.issn.0253-9624.2017.12.009

[45] Rabarison JH Tempia S Harimanana A Guillebaud J Razanajatovo NH Ratsitorahina M Burden and Epidemiology of Influenza- and Respiratory Syncytial Virus-Associated Severe Acute Respiratory Illness Hospitalization in Madagascar, 2011-2016. Influenza Other Respir Viruses. 2019;13:138-47. 10.1111/irv.12557 30596225 PMC6379640

[46] Thapa B Roguski K Azziz-Baumgartner E Siener K Gould P Jamtsho T The Burden of Influenza-Associated Respiratory Hospitalizations in Bhutan, 2015-2016. Influenza Other Respir Viruses. 2019;13:28-35. 10.1111/irv.12605 30137672 PMC6304319

[47] Babakazo P Lubula L Disasuani W Manya LK Nkwembe E Mitongo N The National and Provincial Burden of Medically-Attended Influenza-Associated Influenza-Like-Illness and Severe Acute Respiratory Illness in the Democratic Republic of Congo, 2013-2015. Influenza Other Respir Viruses. 2018;12:695-705. 10.1111/irv.12601 30120818 PMC6185889

[48] Wang X Li Y O’Brien KL Madhi SA Widdowson M-A Byass P Global burden of respiratory infections associated with seasonal influenza in children under 5 years in 2018: a systematic review and modelling study. Lancet Glob Health. 2020;8:e497-510. 10.1016/S2214-109X(19)30545-5 32087815 PMC7083228

[49] GBD 2017 Influenza Collaborators Mortality, morbidity, and hospitalisations due to influenza lower respiratory tract infections, 2017: an analysis for the Global Burden of Disease Study 2017. Lancet Respir Med. 2019;7:69-89. 10.1016/S2213-2600(18)30496-X 30553848 PMC6302221

