# Supplementary material for: Variability in published rates of influenza-associated hospitalizations: A systematic review, 2007-2018
Source: J Glob Health. 2020 Aug 24;10(2):020430. doi: 10.7189/jogh.10.020430 (PMC7699004; doi:10.7189/jogh.10.020430)
Supplement: Online Supplementary Document [file jogh-10-020430-s001.pdf]

Supplementary Document for Variability in Published Rates of Influenza-Associated Hospitalizations: A Systematic Review, 2007–2018

Table S1. Search strategy used across six databases.

Database	Strategy
**Medline (OVID) 1946-**	*Influenza, Human/ OR (influenza* OR acute respiratory infection*).ti<br>AND<br>"Cost of Illness"/ OR *Incidence/ OR Hospitalization/ OR burden.kf,ti. OR hospital*.ti,kf. OR ((cost* OR burden) ADJ3 (illness OR disease OR influenza)).ti,ab.<br>AND<br>Limit yr="2007 – current"; Humans
**Embase (OVID) 1947-**	*Influenza/ OR (influenza* OR acute respiratory infection*).ti<br>AND<br>"Cost of Illness"/ OR Incidence/ OR Hospitalization/ OR burden.kw,ti. OR hospital*.ti,kw. OR ((cost* OR burden) ADJ3 (illness OR disease OR influenza)).ti,ab.<br>AND<br>Limit yr="2007 – current"; Humans; Exclude Medline Journals
**CINAHL (Ebsco) 1982-**	(MJ "Influenza, Human") OR (TI (influenza* OR "acute respiratory infection*"))<br>AND<br>(MH "Cost of Illness") OR (MH Incidence) OR (MH Hospitalization) OR (TI (burden OR surveillance OR hospital* OR ((cost* OR burden) N3 (illness OR disease OR influenza)))) OR (MW (burden OR surveillance OR hospital* OR ((cost* OR burden) N3 (illness OR disease OR influenza))))<br>AND<br>Limit yr="2007 – current"; Humans; Exclude Medline Records
**Cochrane Library 1800-**	[mh "Influenza, Human"] OR (influenza* OR "acute respiratory infection*"):ti<br>AND<br>[mh "Cost of Illness"] OR [mh Incidence] OR [mh Hospitalization] OR burden:ti,ab OR hospital*:ti,ab OR ((cost* OR burden) NEAR/3 (illness OR disease OR influenza)):ti,ab<br>AND<br>Limit yr="2007 – current"; Humans
**Global Health (OVID)**	*Influenza/ OR (influenza* OR acute respiratory infection*).ti<br>AND<br>disease incidence/ OR burden.ti. OR hospital*.ti. OR ((cost* OR burden) ADJ3 (illness OR disease OR influenza)).ti,ab.<br>AND<br>Man/<br>Limit yr="2007 – current"; Humans
**LILACS**	Influenza<br>AND<br>Burden OR cost OR hospitalization OR incidence<br>AND<br>2007-current

Table S2. Specific case definitions reported in extracted articles by analytic case definition.

**Case Definition**	**Detailed Definitions Used in Extracted Articles**
ARI* with fever	• Acute respiratory illness with a history of fever or measured fever (≥38°C) and cough, with onset ≤7 days, requiring hospitalization<br>• Acute respiratory illness with a history of fever or measured fever (≥38°C) and cough, with onset ≤10 days, requiring hospitalization<br>• Hospitalized with acute illness onset (≤14 days) with reported or measured fever (≥38°C) and cough<br>• Hospitalized with measured temperature (≥38°C) or self-reported fever with symptoms of cough or sore throat, and shortness of breath or difficulty breathing with onset ≤10 days of hospital admission<br>• Hospitalized with measured temperature (≥38°C) or self-reported fever with symptoms of cough or sore throat, and shortness of breath or difficulty breathing with onset ≤7 days of hospital admission<br>• Measured temperature (≥38°C) and at least one of: new/worsening cough, sore throat, stuffy nose, runny nose<br>• Admitted to hospital for >24 hours with presence of respiratory difficulty, measured fever (≥38°C), cough, and one or more: confinement to bed, thoracic pain, polypnea, or acute respiratory distress syndrome<br>• Hospitalized with measured fever (≥37.3°C) and at least one: cough, sore throat, tachypnea, difficulty breathing, abnormal breath sounds, sputum production, hemoptysis, chest pain, or chest radiograph consistent with pneumonia<br>• Acute respiratory illness with measured fever (≥38°C) and at least one: cough, sore throat, shortness of breath, difficulty breathing, expectoration, cough blood, chest pain, or lung abnormality showed on X-ray film<br>• Hospitalized with subjective fever in previous 21 days with cough or sore throat<br>• Measured fever (≥38°C) or history of fever in previous 7 days and two or more: cough, sore throat, headache, rhinorrhea, limb or joint pain, vomiting or diarrhea
ARI, fever not required	• ICD10†: J00-J22, J44.0<br>• ICD10: J00-J03, J40-J44, J47<br>• ICD9‡: 460-466<br>• ICD9: 460-466, 480-487, 490, 493, 786, 780.6, 381-382<br>• Hospitalized with cough or difficulty breathing with or without fever<br>• Physician diagnosed lower respiratory infection<br>• Hospitalized with any respiratory symptoms (cough, nasal congestion, coryza, dyspnea, wheezing) or nonlocalizing fever<br>• Hospitalized with cough, difficulty breathing, or pleural chest pain with onset in previous 14 days<br>• Acute upper respiratory tract infection (including colds, sinusitis, pharyngitis, tonsillitis, acute otitis media) or acute lower respiratory tract infection (including croup, trachealis, bronchiolitis, virally induced wheezing, asthma, pneumonia)<br>• Hospitalized with clinically suspected respiratory infection<br>• Bronchitis, pharyngitis or pneumonia with or without fever<br>• Hospitalized with two or more: fever, cough, sore throat, sneezing, congestion, rhinorrhea<br>• Presenting with fever/feverishness or cough at hospital<br>• Hospitalized during defined influenza season with fever and/or ARI symptoms
Pneumonia	• Cough or difficulty breathing AND any of following: unable to drink or breastfeed, vomits everything, convulsions, lethargy, or unconsciousness, chest indrawing or stridor in calm child<br>• Hospitalized with cough or difficulty breathing and any one of the following: tachypnea, unable to drink or breastfeed, vomits everything, convulsions, lethargic or unconscious, nasal flaring, grunting, oxygen saturation <90%, chest indrawing, or stridor in a calm child

Case Definition	Detailed Definitions Used in Extracted Articles
Pneumonia (continued)	• Met criteria for the clinical syndromes of severe pneumonia (cough or difficulty breathing with lower chest indrawing and no signs of very sever pneumonia) OR very sever pneumonia (severe criteria plus hypoxia (O1<90%), inability to drink, inability to sit, and/or impaired consciousness)<br>• Acute illness with one or more of: measured fever ≥38°C, new or worsening cough, dyspnea, consistent auscultation findings [rales or diminished breath sounds], pain in the chest or abdomen when breathing, or purulent or blood-stained sputum production; and radiologic findings consistent with pneumonia
Influenza	• ICD10: J10-J11<br>• ICD10: J10<br>• ICD10: J09-J10<br>• ICD9: 487<br>• Hospitalized with laboratory confirmed influenza infection<br>• Admitted to hospital ≤14 days after or ≤3 days before positive influenza test<br>• Hospitalized for community-onset laboratory confirmed influenza infection<br>• Any patient with clinical features compatible with influenza, requiring hospitalization for clinical severity with at least one: pneumonia, septic shock, acute respiratory distress syndrome, multiple organ dysfunction syndrome, or admission to ICU
Pneumonia and influenza	• ICD10: J09-J18<br>• ICD10: J10-J18<br>• ICD10: J09-J18, J20-22, J28-29, J96, G05.1F. G05.1O, H67.1B, R56.0<br>• ICD9: 480-488<br>• ICD9: 480-487
Respiratory	• ICD10: J00-J99<br>• ICD10: J00-J99, R05-R06, A40-A41, P36<br>• ICD10: J00-J99, H65, H66, H67.1<br>• ICD9: 460-519<br>• ICD9: 460-496<br>• ICD9: 460-519, 079, 786.0-786.4, 786.7-786.9
Circulatory	• ICD10: I00-I99<br>• ICD10: I20-I25, I30, I32, I40-43, I46, I48, I50<br>• ICD9: 380-459<br>• ICD9: 410-414, 420, 422, 425, 477.3, 427.5, 428
Respiratory and circulatory	• ICD10: I00-I99, J00-J99<br>• ICD9: 390-519
Acute medical illness	• ICD10: A00-N99, R00-R99<br>• ICD9: 001-629, 680-739, 780-799<br>• Hospitalized with a medical illness including acute exacerbations of chronic medical conditions (excludes: elective surgery, trauma, obstetrics and gynecologic, orthopedic, ophthalmologic, and psychiatric conditions)
All cause	• ICD10: A00-Y99<br>• ICD9: 001-999
Sepsis	• ICD9: 038, 771.81

*ARI: Acute Respiratory Infection

†ICD10: International Classification of Diseases, 10th Revision

‡ICD9: International Classification of Diseases, 9th Revision

Table S3. Description of extracted articles.

First Author Last Name (Year)	Title	Source	City/State/Country	WHO Region
Abdel-Hady (2018)	Estimating the burden of influenza-associated hospitalization and deaths in Oman (2012-2015)	Influenza and Other Respiratory Viruses 12(1): 146-152.	Oman	Middle East
Adhin (2013)	Trends of influenza infection in Suriname	Influenza Other Respir Viruses 7(5): 629-633.	Suriname	Americas
Ajayi-Obe (2008)	Influenza A and respiratory syncytial virus hospital burden in young children in East London	Epidemiol Infect 136(8): 1046-1058.	East London, UK	Europe
Al-Awaidy (2015)	The Burden of Influenza-Associated Hospitalizations in Oman, January 2008-June 2013	PLoS One 10(12): e0144186.	Oman	Middle East
Ang (2014)	Influenza-associated hospitalizations, Singapore, 2004-2008 and 2010-2012	Emerg Infect Dis 20(10): 1652-1660.	Singapore	Western Pacific
Ang (2017)	Influenza-Associated Hospitalizations for Cardiovascular Diseases in the Tropics	Am J Epidemiol: 1-8.	Singapore	Western Pacific
Appiah (2015)	Influenza activity - United States, 2014-15 season and composition of the 2015-16 influenza vaccine	MMWR Morb Mortal Wkly Rep 64(21): 583-590.	USA	Americas
Azziz-Baumgartner (2012)	Incidence of influenza-like illness and severe acute respiratory infection during three influenza seasons in Bangladesh, 2008-2010	Bull World Health Organ 90(1): 12-19.	Bangladesh	South East Asia
Azziz-Baumgartner (2013)	Incidence of influenza-associated mortality and hospitalizations in Argentina during 2002-2009	Influenza Other Respir Viruses 7(5): 710-717.	Argentina	Americas
Berkley (2010)	Viral etiology of severe pneumonia among Kenyan infants and children	Jama 303(20): 2051-2057.	Kenya	Africa
Blanton (2017)	Update: Influenza Activity in the United States During the 2016-17 Season and Composition of the 2017-18 Influenza Vaccine	MMWR Morb Mortal Wkly Rep 66(25): 668-676.	USA	Americas
Boddington (2017)	Developing a system to estimate the severity of influenza infection in England: findings from a hospital-based surveillance system between 2010/2011 and 2014/2015	Epidemiol Infect 145(7): 1461-1470.	England	Europe
Bolotin (2012)	A new sentinel surveillance system for severe influenza in England shows a shift in age distribution of hospitalised cases in the post-pandemic period	PLoS One 7(1): e30279.	England	Europe
Bonmarin (2015)	Intensive care unit surveillance of influenza infection in France: the 2009/10 pandemic and the three subsequent seasons	Euro Surveill 20(46).	France	Europe

First Author Last Name (Year)	Title	Source	City/State/Country	WHO Region
Bourgeois (2009)	Relative impact of influenza and respiratory syncytial virus in young children	Pediatrics 124(6): e1072-1080.	Massachusetts, USA	Americas
Brammer (2013)	Influenza activity--United States, 2012-13 season and composition of the 2013-14 influenza vaccine	MMWR Morb Mortal Wkly Rep 62(23): 473-479.	USA	Americas
Bresee (2013)	Estimated influenza illnesses and hospitalizations averted by influenza vaccination - United States, 2012-13 influenza season	MMWR Morb Mortal Wkly Rep 62(49): 997-1000.	USA	Americas
Buda (2008)	Epidemiologie der Influenza bei Kindern in Deutschland	Padiatrische Praxis 72: 105-113.	Germany	Europe
Bundy (2010)	Burden of influenza-related hospitalizations among children with sickle cell disease	Pediatrics 125(2): 234-243.	CA, FL, MD, and NY, USA	Americas
Chadha (2013)	Burden of seasonal and pandemic influenza-associated hospitalization during and after 2009 A(H1N1)pdm09 pandemic in a rural community in India	PLoS One 8(5): e55918.	Vadu, India	South East Asia
Chandrasekhar (2017)	Social determinants of influenza hospitalization in the United States	Influenza Other Respir Viruses 11(6): 479-488.	USA	Americas
Chaves (2013)	Patients hospitalized with laboratory-confirmed influenza during the 2010-2011 influenza season: exploring disease severity by virus type and subtype	J Infect Dis 208(8): 1305-1314.	USA	Americas
Chaves (2014)	The burden of influenza hospitalizations in infants from 2003 to 2012, United States	Pediatr Infect Dis J 33(9): 912-919.	USA	Americas
Choi (2017)	Disease burden of 2013-2014 seasonal influenza in adults in Korea	PLoS One 12(3): e0172012.	South Korea	Western Pacific
Cohen (2010)	Trends for influenza and pneumonia hospitalization in the older population: age, period, and cohort effects	Epidemiol Infect 138(8): 1135-1145.	USA	Americas
Cohen (2011)	Influenza vaccination in young children reduces influenza-associated hospitalizations in older adults, 2002-2006	J Am Geriatr Soc 59(2): 327-332.	USA	Americas
Cohen (2013)	Severe influenza-associated respiratory infection in high HIV prevalence setting, South Africa, 2009-2011	Emerg Infect Dis 19(11): 1766-1774.	South Africa	Africa
Cromer (2014)	The burden of influenza in England by age and clinical risk group: a statistical analysis to inform vaccine policy	J Infect 68(4): 363-371.	England	Europe
Dao (2010)	Adult hospitalizations for laboratory-positive influenza during the 2005-2006 through 2007-2008 seasons in the United States	J Infect Dis 202(6): 881-888.	USA	Americas

**First Author Last Name (Year)**	**Title**	**Source**	**City/State/Country**	**WHO Region**
Davila-Torres (2015)	Intense seasonal A/H1N1 influenza in Mexico, winter 2013-2014	Arch Med Res 46(1): 63-70.	Mexico	Americas
Dawa (2018)	National burden of hospitalized and non-hospitalized influenza-associated severe acute respiratory illness in Kenya, 2012-2014	Influenza and Other Respiratory Viruses 12(1): 30-37.	Kenya	Africa
Dawood (2010)	Burden of seasonal influenza hospitalization in children, United States, 2003 to 2008	J Pediatr 157(5): 808-814.	USA	Americas
D'Onise (2008)	The burden of influenza in healthy children in South Australia	Med J Aust 188(9): 510-513.	South Australia	Western Pacific
Emukule (2014)	The burden of influenza and RSV among inpatients and outpatients in rural western Kenya, 2009-2012	PLoS One 9(8): e105543.	Western Kenya	Africa
Feikin (2012)	The population-based burden of influenza-associated hospitalization in rural western Kenya, 2007-2009	Bull World Health Organ 90(4): 256-263a.	Western Kenya	Africa
Feng (2014)	[Influenza-associated-excess-hospitalization in children, Wuxi city, Jiangsu province, 2005-2010]	Zhonghua Liu Xing Bing Xue Za Zhi 35(6): 699-703.	Wuxi city, Jiangsu province, China	Western Pacific
Fry (2014)	Modeling the effect of different vaccine effectiveness estimates on the number of vaccine-prevented influenza-associated hospitalizations in older adults	Clin Infect Dis 59(3): 406-409.	USA	Americas
Fuller (2013)	Estimation of the national disease burden of influenza-associated severe acute respiratory illness in Kenya and Guatemala: a novel methodology	PLoS One 8(2): e56882.	Kenya and Guatemala	Africa and Americas
Ganatra (2012)	Update: influenza activity - United States, 2011-12 season and composition of the 2012-13 influenza vaccine	MMWR Morb Mortal Wkly Rep 61(22): 414-420.	USA	Americas
Gefenaite (2018)	Estimating burden of influenza-associated influenza-like illness and severe acute respiratory infection at public healthcare facilities in Romania during the 2011/12-2015/16 influenza seasons	Influenza and Other Respiratory Viruses 12(1): 183-192.	Romania	Europe
Goldstein (2015)	Estimating the hospitalization burden associated with influenza and respiratory syncytial virus in New York City, 2003-2011	Influenza Other Respir Viruses 9(5): 225-233.	New York City, NY, USA	Americas
Gouya (2016)	Estimation of influenza and severe acute respiratory illness incidence (burden) in three provinces of the Islamic Republic of Iran, 2012 and 2013	East Mediterr Health J 22(7): 432-439.	Iran	Middle East
Grijalva (2007)	Estimating the undetected burden of influenza hospitalizations in children	Epidemiol Infect 135(6): 951-958.	USA	Americas

First Author Last Name (Year)	Title	Source	City/State/Country	WHO Region
Hadler (2014)	Impact of requiring influenza vaccination for children in licensed child care or preschool programs--Connecticut, 2012-13 influenza season	MMWR Morb Mortal Wkly Rep 63(9): 181-185.	CT, USA	Americas
Hardelid (2017)	Risk factors for admission to hospital with laboratory-confirmed influenza in young children: birth cohort study	Eur Respir J 50(3).	Scotland, UK	Europe
Hirve (2015)	Incidence of influenza-associated hospitalization in rural communities in western and northern India, 2010-2012: a multi-site population-based study	J Infect 70(2): 160-170.	Ballabgarh and Vadu, India	South East Asia
Huang (2015)	Southern Hemisphere Influenza and Vaccine Effectiveness Research and Surveillance	Influenza Other Respir Viruses 9(4): 179-190.	New Zealand	Western Pacific
Jacks (2012)	Influenza-associated hospitalisations in Finland from 1996 to 2010: unexpected age-specific burden during the influenza A(H1N1)pdm09 pandemic from 2009 to 2010	Euro Surveill 17(38).	Finland	Europe
Jules (2014)	Age-Specific Influenza-Related Emergency Department Visits and Hospitalizations in 2010-2011 Compared With the Pandemic Year 2009-2010	Infectious Diseases in Clinical Practice 22(5): 271-278.	TN, USA	Americas
Jules (2015)	Influenza-related hospitalization and ED visits in children less than 5 years: 2000-2011	Pediatrics 135(1): e66-74.	Davidson County, TN, USA	Americas
Kamigaki (2017)	Estimates of influenza and respiratory syncytial virus incidences with fraction modeling approach in Baguio City, the Philippines, 2012-2014	Influenza Other Respir Viruses 11(4): 311-318.	Baguio City, the Philippines	Western Pacific
Khieu (2015)	Estimating the contribution of influenza to hospitalisations in New Zealand from 1994 to 2008	Vaccine 33(33): 4087-4092.	New Zealand	Western Pacific
Kniss (2011)	Update: influenza activity--United States, 2010-11 season, and composition of the 2011-12 influenza vaccine	MMWR Morb Mortal Wkly Rep 60(21): 705-712.	USA	Americas
Kostova (2013)	Influenza Illness and Hospitalizations Averted by Influenza Vaccination in the United States, 2005-2011	PLoS One 8(6): e66312.	USA	Americas
Le (2014)	Impact of 2009 pandemic influenza among Vietnamese children based on a population-based prospective surveillance from 2007 to 2011	Influenza Other Respir Viruses 8(4): 389-396.	Nha Trang city, Vietnam	Western Pacific
Matias (2016)	Modelling estimates of age-specific influenza-related hospitalisation and mortality in the United Kingdom	BMC Public Health 16(1): 481.	United Kingdom	Europe

First Author Last Name (Year)	Title	Source	City/State/Country	WHO Region
Matias (2017)	Estimates of hospitalization attributable to influenza and RSV in the US during 1997-2009, by age and risk status	BMC Public Health 17(1): 271.	USA	Americas
McMorrow (2015)	The Unrecognized Burden of Influenza in Young Kenyan Children, 2008-2012	PLoS One 10(9): e0138272.	Kenya	Africa
Millman (2015)	Improving Accuracy of Influenza-Associated Hospitalization Rate Estimates	Emerg Infect Dis 21(9): 1595-1601.	USA	Americas
Murray (2015)	Determining the Provincial and National Burden of Influenza-Associated Severe Acute Respiratory Illness in South Africa Using a Rapid Assessment Methodology	PLoS One 10(7): e0132078.	South Africa	Africa
Nyamusore (2018)	The national burden of influenza-associated severe acute respiratory illness hospitalization in Rwanda, 2012-2014	Influenza and Other Respiratory Viruses 12(1): 38-45.	Rwanda	Africa
Oliva (2018)	Estimating the burden of seasonal influenza in Spain from surveillance of mild and severe influenza disease, 2010-2016	Influenza and Other Respiratory Viruses 12(1): 161-170.	Spain	Europe
Poehling (2013)	The burden of influenza in young children, 2004-2009	Pediatrics 131(2): 207-216.	USA	Americas
Preaud (2014)	Annual public health and economic benefits of seasonal influenza vaccination: a European estimate	BMC Public Health 14: 813.	France, Germany, the UK, Spain, Italy, Sweden, Poland, and Slovakia	Europe
Proff (2009)	Case-based surveillance of influenza hospitalizations during 2004-2008, Colorado, USA	Emerg Infect Dis 15(6): 892-898.	Colorado, USA	Americas
Puig-Barbera (2012)	Effectiveness of the 2010-2011 seasonal influenza vaccine in preventing confirmed influenza hospitalizations in adults: a case-case comparison, case-control study	Vaccine 30(39): 5714-5720.	Valencia, Spain	Europe
Rajaram (2017)	Impact of increased influenza vaccination in 2-3-year-old children on disease burden within the general population: A Bayesian model-based approach	PLoS One 12(12): e0186739.	England	Europe
Reed (2014)	Estimated influenza illnesses and hospitalizations averted by vaccination--United States, 2013-14 influenza season	MMWR Morb Mortal Wkly Rep 63(49): 1151-1154.	USA	Americas
Reed (2015)	Estimating influenza disease burden from population-based surveillance data in the United States	PLoS One 10(3): e0118369.	USA	Americas

First Author Last Name (Year)	Title	Source	City/State/Country	WHO Region
Refaey (2016)	Incidence of influenza virus-associated severe acute respiratory infection in Damanhour district, Egypt, 2013	East Mediterr Health J 22(7): 503-512.	Damanhour district, Egypt	Middle East
Rodrigues (2018)	Excess pneumonia and influenza hospitalizations associated with influenza epidemics in Portugal from season 1998/1999 to 2014/2015	Influenza and Other Respiratory Viruses 12(1): 153-160.	Portugal	Europe
Sakkou (2011)	Impact of influenza infection on children's hospital admissions during two seasons in Athens, Greece	Vaccine 29(6): 1167-1172.	Athens, Greece	Europe
Schanzer (2008)	Role of influenza and other respiratory viruses in admissions of adults to Canadian hospitals	Influenza Other Respir Viruses 2(1): 1-8.	Canada	Americas
Schanzer (2013)	Statistical estimates of respiratory admissions attributable to seasonal and pandemic influenza for Canada	Influenza Other Respir Viruses 7(5): 799-808.	Canada	Americas
Schanzer (2018)	Burden of influenza, respiratory syncytial virus, and other respiratory viruses and the completeness of respiratory viral identification among respiratory inpatients, Canada, 2003-2014	Influenza and Other Respiratory Viruses 12(1): 113-121.	Canada	Americas
Sheu (2016)	Comparison of age-specific hospitalization during pandemic and seasonal influenza periods from 2009 to 2012 in Taiwan: a nationwide population-based study	BMC Infect Dis 16: 88.	Taiwan	Western Pacific
Sloan (2015)	Socioeconomic Disparities and Influenza Hospitalizations, Tennessee, USA	Emerg Infect Dis 21(9): 1602-1610.	TN, USA	Americas
Sotomayor (2018)	Estimating the burden of influenza-associated hospitalizations and deaths in Chile during 2012-2014	Influenza and Other Respiratory Viruses 12(1): 138-145.	Chile	Americas
Stewart (2018)	Using a hospital admission survey to estimate the burden of influenza-associated severe acute respiratory infection in one province of Cambodia—methods used and lessons learned	Influenza and Other Respiratory Viruses 12(1): 104-112.	Cambodia	Western Pacific
Susilarini (2018)	Estimated incidence of influenza-associated severe acute respiratory infections in Indonesia, 2013-2016	Influenza and Other Respiratory Viruses 12(1): 81-87.	Indonesia	South East Asia
Talbot (2009)	Influenza in older adults: impact of vaccination of school children	Vaccine 27(13): 1923-1927.	TN, USA	Americas
Tallo (2014)	Estimating influenza outpatients' and inpatients' incidences from 2009 to 2011 in a tropical urban setting in the Philippines	Influenza Other Respir Viruses 8(2): 159-168.	Philippines	Western Pacific

First Author Last Name (Year)	Title	Source	City/State/Country	WHO Region
Tam (2014)	Influenza-related hospitalization of adults associated with low census tract socioeconomic status and female sex in New Haven County, Connecticut, 2007-2011	Influenza Other Respir Viruses 8(3): 274-281.	New Haven County, CT, USA	Americas
Taylor (2017)	Respiratory viruses and influenza-like illness: Epidemiology and outcomes in children aged 6 months to 10 years in a multi-country population sample	J Infect 74(1): 29-41.	Brazil, Colombia, Costa Rica, Mexico, Philippines, Singapore, Thailand	Americas, South East Asia, Western Pacific
Tempia (2018)	The effects of the attributable fraction and the duration of symptoms on burden estimates of influenza-associated respiratory illnesses in a high HIV prevalence setting, South Africa, 2013-2015	Influenza Other Respir Viruses 12(3): 360-373.	South Africa	Africa
Theo (2018)	The national burden of influenza-associated severe acute respiratory illness hospitalization in Zambia, 2011-2014	Influenza and Other Respiratory Viruses 12(1): 46-53.	Zambia	Africa
Tinoco (2017)	Burden of Influenza in 4 Ecologically Distinct Regions of Peru: Household Active Surveillance of a Community Cohort, 2009-2015	Clin Infect Dis 65(9): 1532-1541.	Peru	Americas
von der Beck (2017)	Characteristics and outcomes of a cohort hospitalized for pandemic and seasonal influenza in Germany based on nationwide inpatient data	PLoS One 12(7): e0180920.	Germany	Europe
Widmer (2012)	Rates of hospitalizations for respiratory syncytial virus, human metapneumovirus, and influenza virus in older adults	J Infect Dis 206(1): 56-62.	Davidson County, TN, USA	Americas
Wong (2009)	Influenza-associated hospitalisation	Hong Kong Med J 15 Suppl 9: 35-37.	Hong Kong	Western Pacific
Wu (2017)	A joint analysis of influenza-associated hospitalizations and mortality in Hong Kong, 1998-2013	Sci Rep 7(1): 929.	Hong Kong	Western Pacific
Yoshida (2010)	Viral pathogens associated with acute respiratory infections in central vietnamese children	Pediatr Infect Dis J 29(1): 75-77.	Vietnam	Western Pacific
Yousey-Hindes (2011)	Neighborhood socioeconomic status and influenza hospitalizations among children: New Haven County, Connecticut, 2003-2010	Am J Public Health 101(9): 1785-1789.	New Haven County, CT, USA	Americas
Yu (2014)	The substantial hospitalization burden of influenza in central China: surveillance for severe, acute respiratory infection, and influenza viruses, 2010-2012	Influenza Other Respir Viruses 8(1): 53-65.	Jingzhou, China	Western Pacific
Zhang (2017)	Pneumonia and influenza hospitalizations among children under 5 years of age in Suzhou, China, 2005-2011	Influenza Other Respir Viruses 11(1): 15-22.	Suzhou, China	Western Pacific

First Author Last Name (Year)	Title	Source	City/State/Country	WHO Region
Zhang (2017)	[Estimating the burden of influenza-associated hospitalization for cases of severe acute respiratory infection, Beijing, 2015]	Zhonghua Yu Fang Yi Xue Za Zhi 51(12): 1097-1101.	Beijing, China	Western Pacific
Zheng (2015)	[Estimation of hospitalization rate of laboratory confirmed influenza cases in Jingzhou city, Hubei province, 2010-2012]	Zhonghua Liu Xing Bing Xue Za Zhi 36(3): 222-227.	Jingzhou city, Hubei province, China	Western Pacific
Zhou (2012)	Hospitalizations associated with influenza and respiratory syncytial virus in the United States, 1993-2008	Clin Infect Dis 54(10): 1427-1436.	USA	Americas

Table S4. Variance attributed to study heterogeneity (I^2) within each study characteristic category.

**Category**	**I^2**
Age Group	
• All Age	99.93%
• < 2 Years	99.98%
• 2 – 4 Years	99.99%
• < 5 Years	99.95%
• 5 – 14 Years	99.82%
• 15 – 49 Years	99.80%
• 50 – 64 Years	99.56%
• ≥ 65 Years	99.90%
Method	
• Multiplier	99.97%
• Regression Modelling	99.97%
Case-patient identification method	
• Symptom-based identification	99.97%
• Administrative database	99.95%
World Bank income grouping	
• High	99.95%
• Upper-middle	99.99%
• Lower-middle	99.17%
• Low	99.92%
World Health Organization region	
• Sub-Saharan Africa	99.89%
• Americas	99.77%
• Eastern Mediterranean	99.61%
• Europe	99.99%
• South-East Asia	99.45%
• Western Pacific	99.99%

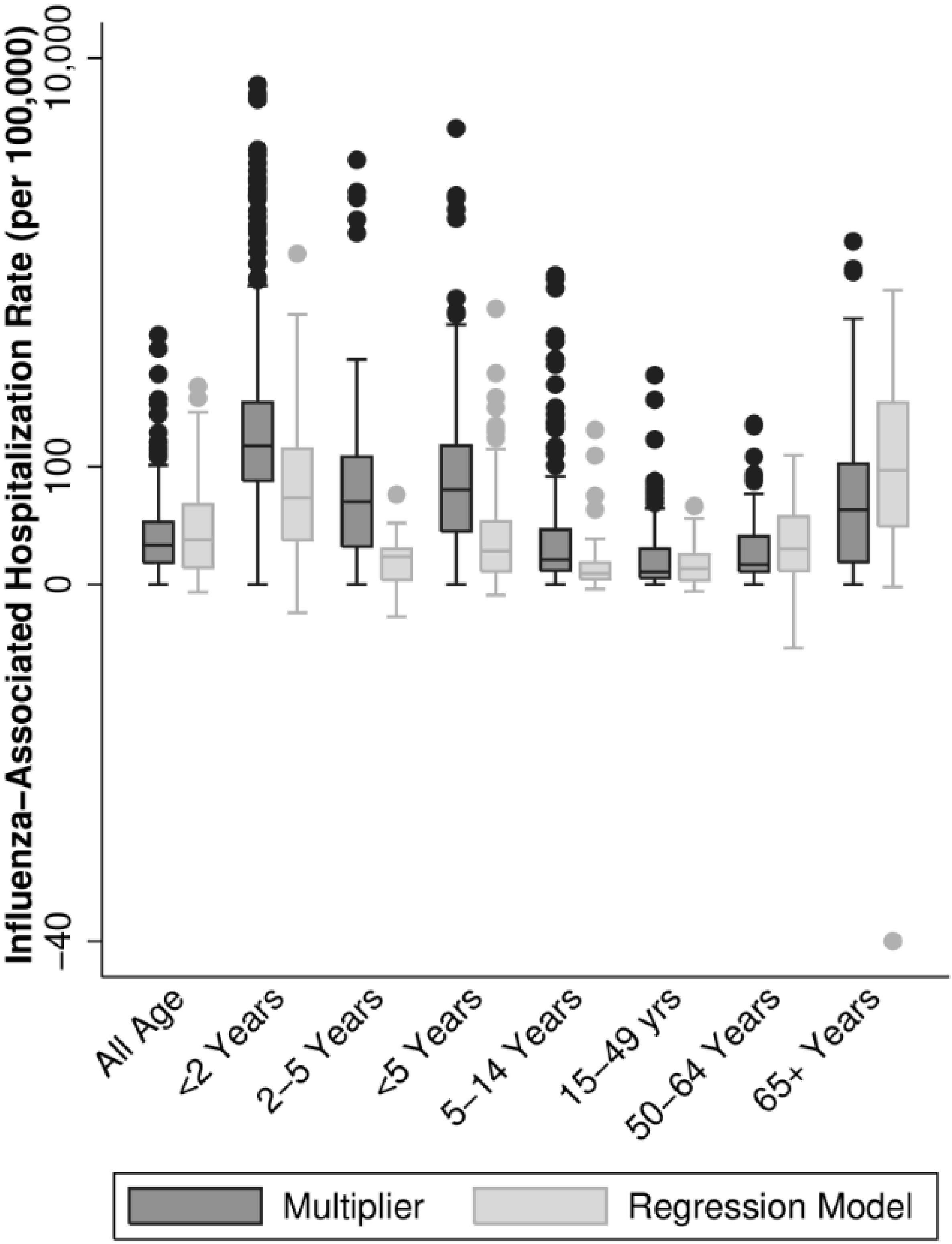

Figure S1. Published rates of influenza-associated hospitalization, by combined age groups and analytic method.
